# Effects of 4-Week Tangeretin Supplementation on Cortisol Stress Response Induced by High-Intensity Resistance Exercise: A Randomized Controlled Trial

**DOI:** 10.3389/fphys.2022.886254

**Published:** 2022-05-19

**Authors:** Meng Liu, Zheng Zhang, Chunli Qin, Bingqiang Lv, Shiwei Mo, Tao Lan, Binghong Gao

**Affiliations:** ^1^ College of Physical Education and Training, Shanghai University of Sport, Shanghai, China; ^2^ Chongqing Institute of Sport Science, Chongqing, China; ^3^ School of Kinesiology, Shanghai University of Sport, Shanghai, China; ^4^ School of Physical Education, Shenzhen University, Shenzhen, China; ^5^ Sports and Art Department, Hebei Sport University, Shijiazhuang, China

**Keywords:** tangeretin, exercise test, cortisol stress responses, resistance exercise, serum cortisol, antioxidant capacity

## Abstract

**Objective:** This study aimed to investigate the effects of 4-week tangeretin supplementation on the cortisol stress response induced by high-intensity resistance exercise.

**Methods:** A randomized controlled trial of twenty-four soccer players was conducted during the winter training season. The experimental group (EG) took the oral supplement with tangeretin (200 mg/day) and the control group (CG) took placebo for 4 weeks. Before and after the 4-week intervention, all players performed a high intensity bout of resistance exercise to stimulate their cortisol stress responses. Serum cortisol, adreno-corticotropic hormone (ACTH) and superoxide dismutase (SOD) were obtained by collecting blood samples before (PRE), immediately after (P0), and 10 (P10), 20 (P20) and 30 minutes (P30) after the exercise.

**Results:** The serum cortisol level (PRE, *p* = 0.017; P10, *p* = 0.010; P20, *p* = 0.014; P30, *p* = 0.007) and ACTH (P10, *p* = 0.037; P30, *p* = 0.049) of experimental group significantly decreased after the 4-week intervention. Compared with control group, EG displayed a significantly lower level of the serum cortisol (PRE, *p* = 0.036; P10, *p* = 0.031) and ACTH (P30, *p* = 0.044). Additionally, EG presented significantly higher superoxide dismutase activity level compared with CG at P30 (*p* = 0.044). The white blood cell of EG decreased significantly (PRE, *p* = 0.037; P30, *p* = 0.046) and was significantly lower than CG at P20 (*p* = 0.01) and P30 (*p* = 0.003).

**Conclusion:** Four-week tangeretin supplementation can reduce serum cortisol and ACTH, which may ameliorate the cortisol stress response in soccer players during high-intensity resistance exercise training. It can also enhance antioxidant capacity, accelerate the elimination of inflammation throughout the body, and shorten recovery time after high-intensity exercise.

## 1 Introduction

The adrenal gland responds when the body is confronted with psychological or physiological stress by releasing cortisol (Simons et al., 2017). The stress response could regulate the metabolism of various energy sources and substances in blood (Viru and Viru, 2004). Although the cortisol response plays an irreplaceable role on maintaining normal physiological function, the overt stress response or chronic elevation of blood cortisol concentration could negatively affect many physiological functions, such as protein metabolism, inflammatory processes and glucose-alanine cycle (Cadegiani and Kater, 2017; Hodes et al., 2018; Walter et al., 2018). Nutritional interventions have been proved valuable for moderately attenuating the cortisol response induced by intense exercise or chronic mental stress in the human body (Kraemer et al., 1998; Córdova et al., 2019; Tsuda et al., 2020). The dietary supplements of plant origin, due to the properties of natural compounds and free of banned substances, have gradually become the prior choice of nutritional interventions for cortisol regulation (Assini et al., 2013; Kioukia-Fougia et al., 2017).

**FIGURE 1:**
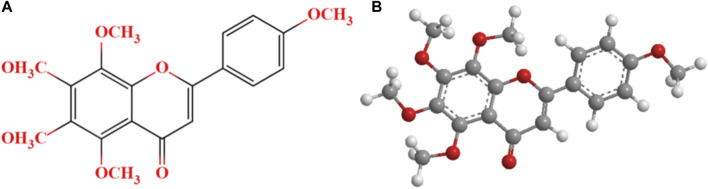
The chemical structure **(A)** and 3D chemical structure **(B)** of tangeretin.

Plant flavonoids, widespreading in fruits and vegetables, are often reported to effectively modulate cortisol concentrations (Ruijters et al., 2014; Szelényi et al., 2019). Tangeretin (TG, Figure 1), a citrus flavonoid extracted from citrus peel, has been proved possessing excellent antioxidant, anti-inflammatory and neuroprotective properties (Liu et al., 2018; Zhao et al., 2018). TG was previously found to enhance the activity of antioxidant enzymes, reduce the level of oxidative stress and dramatically extend the swimming time to exhaustion in mice (Kou et al., 2018; Kou et al., 2019). Further, in our previous study, TG intervention (200 mg/d) for 30 days was detected to significantly increase the maximal oxygen uptake and time to exhaustion in athletes with exercise-induced bronchoconstriction (Liu et al., 2021; Liu and Gao, 2022). The aforementioned studies suggest that TG may possess anti-fatigue property. In another study, we found that blood cortisol and uric acid concentrations significantly decreased in weightlifters after orally taking TG supplementation (200 mg/d) for 5 weeks (Liu et al., 2019). However, it is difficult to determine the role of TG on decrement of blood cortisol concentrations because we did not include a control group and illuminate effects of dietetic food.

The model of acute intense resistance exercise has been proved dramatically increasing blood cortisol concentration (Kraemer and Ratamess, 2005). Increased cortisol concentration is usually associated with anticipation of high-intensity exercise in human beings (Jamurtas et al., 2018; Wang et al., 2019), which indicates that acute intensity resistance exercise may be a suitable method for examining the benefits of TG supplementation. Therefore, this study aimed to determine the effects of TG supplementation on cortisol responses induced by an acute intense resistance exercise, and its potential impacts on adreno-corticotropic hormone (ACTH), superoxide dismutase (SOD) and WBC. We hypothesized that TG supplementation would contribute to regulating cortisol response and reducing blood cortisol concentrations before and after the acute intense resistance exercise.

## 2 Materials and Methods

### 2.1 Participants

**FIGURE 2:**
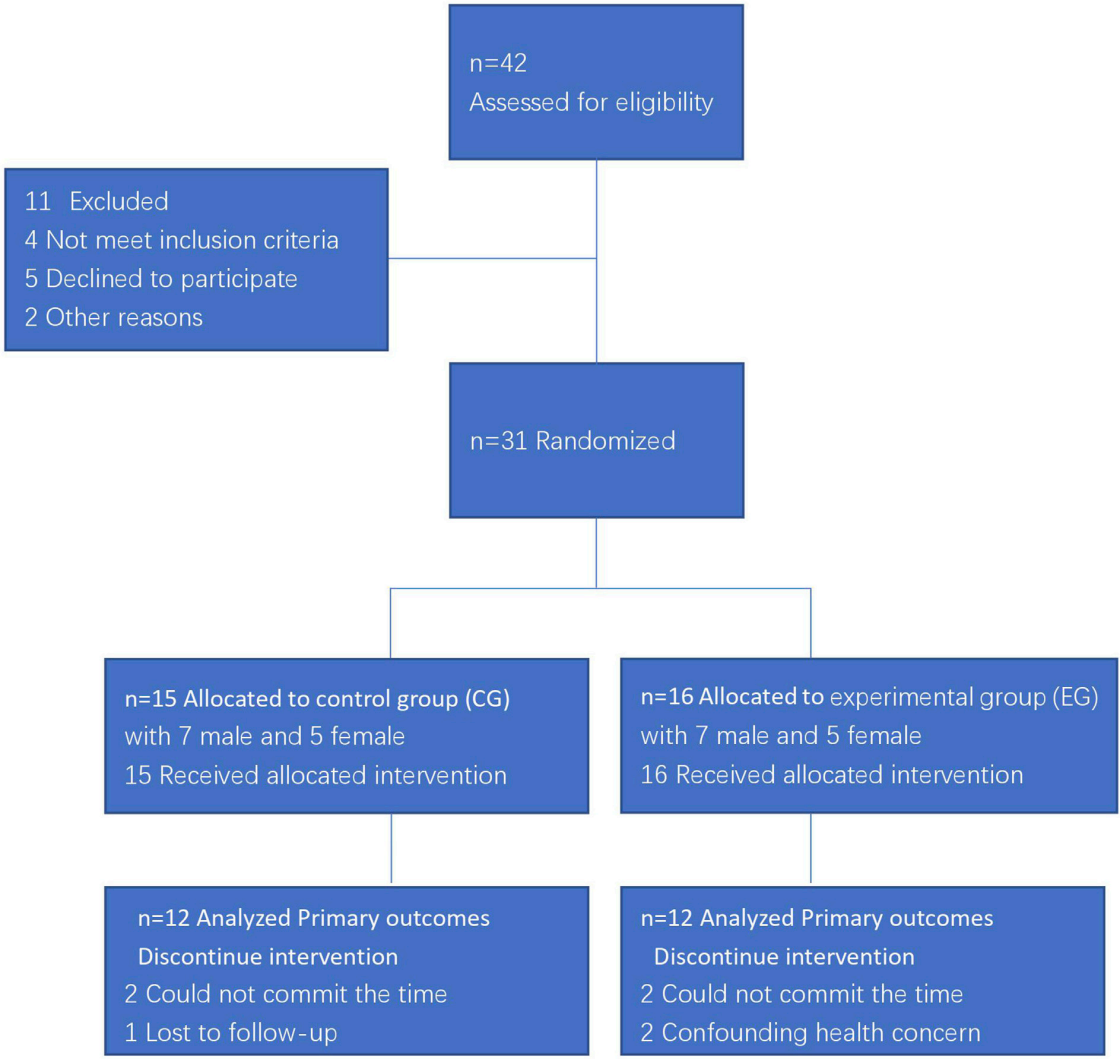
The flow diagram of study.

Participants were recruited from soccer players of the Chongqing Li-Fan professional football club. According to inclusion and exclusion criteria, eligible respondents were randomly grouped into experimental group (EG) or control group (CG). The flow diagram of study was presented in Figure 2.

Twenty-four players (10F/14M) finally completed the experiment in this study. All the players have prior experience of resistance hemolysis (5.3 ± 1.2 years). Their mean (standard deviation, SD) age and height were respectively 20.3 ± 1.2 years and 172.6 ± 6.1 cm. According to one-repetition maximum (1RM) test adopted from the study by Kraemer et al. (2005), their 1RM of bench press, back squat, shoulder press and deadlift were 68.2 ± 24.3 kg, 118.9 ± 38.1 kg, 59.3 ± 22.4 kg and 102.9 ± 30.1 kg, respectively. Additionally, their training program (frequency, duration and intensity) and schedule (training and resting day) were similar throughout the study. Their training daily information is presented in detail in Supplement S1. Each player was informed the experimental procedures, benefits and risks and provided signed written consent before data collection. This study was approved by the Academic and Human Rights Ethics Committee, Shanghai University of Sport.

An *a priori* sample size was estimated through G*Power 3.1 using the effect size data (Cohen’s *d* = 0.79), which was calculated based on the blood cortisol concentration (15.15 ± 4.36 μg/dl vs 12.21 ± 2.91 μg/dl) before and after 4-week intervention in 12 sprinters (Liu et al., 2021). Considering 20% attrition, 13 participants were deemed to sufficient to obtain a desired power of 80% at *α* = 0.05.

### 2.2 Methods

**FIGURE 3:**
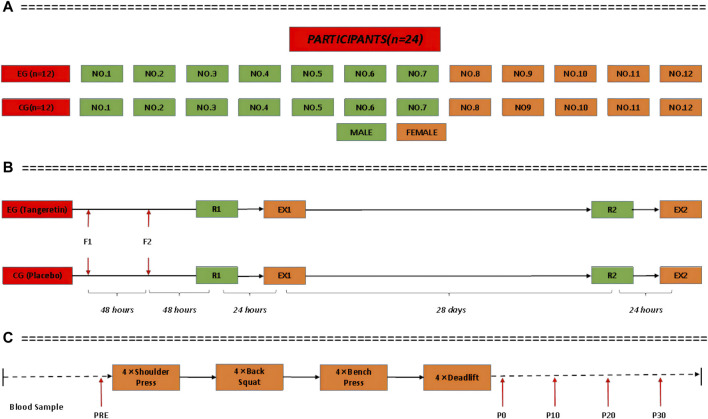
Participants paired according to sex, height, weight and sports level.

A paired, randomized, double-blind experimental protocol was used in this study. All the players were paired according to sex, height, weight and sports level. They were numbered and randomly divided into EG or CG using a computer to generate random numbers, with seven men (NO.1-NO.7) and five women (NO.8-NO.12) in each group (Figure 3A). The players of both EG and CG were instructed to complete the supplement intervention, exercise stimulation test and body composition test. Grouping methods and intervention assignments (tangeretin vs placebo) were blinded to both researchers and the players.

#### 2.2.1 Supplement Intervention

Each day, all the players were required to enter the laboratory in early morning (7:30–8:30 a.m.). Each received a bottle of supplement drink (200 ml). The supplement drink was packed in a sealed opaque glass bottle and prepared in advance by a research assistant, who was blind to the component of the supplement drink. The bottle was labeled using each players’ pseudonym ID. Each player was asked to oral the corresponding supplement drink and was not informed the component of the drink. For EG, the supplement drink was made using a self-developed and commercially available supplement product (Qinguoren tangeretin supplement^®^; China Anti-Doping Center test report number: 2019FD234) referring to 19.8 g whey protein isolate powders (≥95% purity, CanSure, Vancouver, BC, Canada; China Anti-Doping Center test report number: 2019FD279) and 200 mg tangeretin (99.79% purity). The Qingguoren^®^ tangeretin supplement has yet to mass produce and right now can only be purchased from Southwest Institute of Fruits Nutrition. For CG, the supplement drink contained a placebo supplement, consisting of only 20 g whey protein isolate powders. The drinks were identical in terms of aesthetics, weight, and flavor, with the only difference being the presence or omission of tangeretin. The supplement drink was prepared daily by the research assistant.

The dose of 200 mg/day was chose based on evidence from previous studies. Based on findings from previous animal experiment (Nakajima et al., 2020), daily intaking tangeretin for 1–5 mg per kilogram bodyweight did not cause any side effects. Other studies (Hu et al., 2020; Kou et al., 2018; 2019) detected certain benefits when daily intaking tangeretin supplementation for 3–4 mg per kilogram bodyweight. Moreover, our preliminary human trial studies (Liu et al., 2019; 2021) indicated that athletes orally taking tangeretin supplementation for 200 mg per day for 5 weeks or 30 days improved their physical function (i.e., serum testosterone, cortisol, etc.) and was free of any uncomfortable symptoms (i.e., dizziness, vomiting, etc.).

Throughout the study period (a total of 43 days), all players only consumed food, including snacks and fruit, which was supplied by the Chongqing Competitive Sports Training Center. To minimize effects of food and condiment (Cheng and Li, 2012), dietary advice was provided and ingredient selection was monitored. Except the Gatorade sports drinks, all players were also prohibited from any other supplement, including traditional Chinese medicine. All players reported no signs or symptoms of discomfort throughout the study period.

#### 2.2.2 Exercise Stimulation Experiment

For the cortisol stress response provocation test, each player was instructed to complete a standard high-intensity resistance exercise (EX) protocol on the day before and after the intervention (Figure 3B). The protocol consisted of four weightlifting (shoulder press, back squat, bench press and deadlift), four sets each weightlifting, and interval of 2 min for rest (Figure 3C). For each player, the weight for each lift was equal to his/her 10RM (the maximum weight allowing to perform a lift for only ten repetitions per time). The 10RM was predetermined for each player during the familiarization session (F) using a standard protocol adopted from the study by Kraemer et al. (2005). The method was confirmed to be reliable with intra-class correlation coefficient being 0.98 for two consecutive exercise sessions (Figure 3B). A professional coach provided supervision throughout the weightlifting to maximum each players’ cortisol stress response and to minimize the risk of injury. All the players were allowed to consume water as well as some snacks if required.

The goal of the familiarizations was to create a reliable workout that could be highly replicated to produce a similar (if not identical) physiological stress response. During the experimental workout, resistances were reduced to allow only 10 repetitions to be performed but both experimental workouts used very similar (if not identical) resistances in the workout sequences because of careful familiarization and practices of the experimental protocol (Kraemer et al., 1990).

Blood samples were collected immediately before (PRE, immediately before the exercise) and after each provocation test (Figure 3C). Sampling took place between 2:00 p.m. and 5:00 p.m. A 3 ml sample of venous blood was taken each time (tube A: 1 ml, tube B: 2 ml). Blood samples were collected at four time points after the provocation test: immediately (P0, immediately after the exercise), 10 min (P10), 20 min (P20), and 30 min (P30) (Figure 3C). The white blood cell (WBC) level of tube A samples was measured with a hematology analyzer (Mindray BC-5150, China) at 10 min after collection. Serum samples were separated from tube B blood samples on a high-speed centrifuge (Shuke TG16, China) at 2000 R/min for 15 min, within 30 min after collection, and stored at −80°C in a medical freezer (Boke BDF-86V158, China). Analysis was performed at the end of the provocation test by a researcher (Mindray SAL-6000, China; BioTek-Epoch, United States). Serum cortisol, superoxide dismutase (SOD), adreno-corticotropic hormone (ACTH), and WBC were measured and recorded. In addition, the blood lactate level of each athlete was measured with a portable blood lactate analyzer (EKF, Germany) at the PRE and P0 time points for each provocation test. In order to collect more accurate data from blood samples, the appearance of the sample was first qualitatively ranked by trained laboratory technologists from “no visible hemolysis” to “4 + hemolysis,” and its hemoglobin concentration was determined by the benzidine method (Crosby et al., 1954).

#### 2.2.3 Body Composition Test

Body composition was measured for all players before (R1) and after (R2) the intervention using a multi-frequency bioelectrical impedance analyzer (InBody^®^ 570, BioSpace Inc, Seoul, Korea), see Figure 3B. This method was validated using dual-energy x-ray absorptiometry and confirmed to be valid and reliable (Miller et al., 2016). During the test, each player was instructed to wear light clothes and statically stand on the analyzer in barefoot. Body weight, muscle mass and body fat percentage were obtained and analyzed.

### 2.3 Statistical Analysis

Statistical analysis was conducted with SPSS 25.0 package. The results were showed as mean ± SD.

Following check for normality (Shapiro-Wilk), sphericity of each dataset (Mauchly’s test), and homogeneity of variance between groups (Levene’s test), the 10RM values of athletes in the four resistance exercise sessions (shoulder press, back squat, bench press, and deadlift) and various biochemical indices of the cortisol stress response (serum cortisol, SOD, ACTH, WBC and blood lactate) were analyzed with two-way analysis of variance (ANOVA) with repeated measurements (2 groups × different time points). If a significant difference was indicated, pairwise comparison was carried using the least significance difference (LSD) test. The significance level was set at *p* < 0.05.

## 3 Results

### 3.1 Comparison of Body Composition

There were no statistical differences in body weight, body fat percentage and muscle mass before and after the intervention in both group (Table 1).

**TABLE 1:** Effects of tangeretin intervention on body composition.

	Body weight (kg)		Body fat percentage (%)		Muscle Mass (kg)	
	R1	R2	R1	R2	R1	R2
**EG**	61.8 ± 6.0	61.9 ± 5.9	13.3 ± 4.4	12.9 ± 3.7	30.5 ± 4.3	30.8 ± 4.3
**CG**	59.1 ± 9.8	59.3 ± 10.3	12.9 ± 4.7	13.1 ± 4.6	28.6 ± 5.3	28.9 ± 5.7
**Main effect - Time**	*p* = 0.408; η^2^ = 0.063		*p* = 0.665; η^2^ = 0.020		*p* = 0.353; η^2^ = 0.087	
**Main effect - Group**	*p* = 0.448; η^2^ = 0.053		*p* = 0.459; η^2^ = 0.056		*p* = 0.050; η^2^ = 0.332	
**Interaction - Time× Group**	*p* = 0.921; η^2^ = 0.001		*p* = 0.115; η^2^ = 0.229		*p* = 0.869; η^2^ = 0.003	

CG: control group; EG: experimental group

### 3.2 Effects on 10RM of Resistance Exercises and Blood Lactate

The 10RM values of the four resistance exercises (shoulder press, back squat, bench press, and deadlift) before and after the intervention were presented in Table 2. There were no group differences before and after the intervention. Although the 10RM values of both groups increased slightly after the intervention, the difference was not statistically significant. There were also no statistical differences in the level of blood lactate between EG and CG before and after the intervention. The level of blood lactate increased significantly in both groups after the high-intensity resistance exercise (Table 3).

**TABLE 2:** Comparison of 10RM of the back squat, bench press, deadlift, and shoulder press (kg).

	Shoulder Press（kg）		Back Squat（kg）		Bench Press（kg）		Deadlift（kg）	
	T1	T2	T1	T2	T1	T2	T1	T2
**EG**	54.3 ± 19.1	58.2 ± 21.8	91.1 ± 22.4	93.2 ± 23.5	49.6 ± 17.2	52.9 ± 16.4	93.2 ± 22.7	94.3 ± 23.2
**CG**	53.6 ± 18.2	54.6 ± 17.9	89.6 ± 21.7	92.1 ± 22.3	48.2 ± 16.2	50.7 ± 15.1	95.4 ± 23.6	97.1 ± 25.2
**Main effect - Time**	*p* = 0.396; η^2^ = 0.056		*p* = 0.110; η^2^ = 0.184		*p* = 0.065; η^2^ = 0.238		*p* = 0.518; η^2^ = 0.033	
**Main effect - Group**	*p* = 0.029; η^2^ = 0.318		*p* = 0.138; η^2^ = 0.161		*p* = 0.001; η^2^ = 0.610		*p* = 0.055; η^2^ = 0.254	
**Interaction - Time × Group**	*p* = 0.241; η^2^ = 0.104		*p* = 0.583; η^2^ = 0.024		*p* = 0.165; η^2^ = 0.143		*p* = 0.612; η^2^ = 0.020	

CG: control group; EG: experimental group. T1: Before 4-week tangeretin intervention; T2: After 4-week tangeretin intervention

**TABLE 3:** Comparison of blood lactate before and after high-intensity resistance exercise test (mmol/L).

	1st High-Intensity Resistance Exercise		2nd High-Intensity Resistance Exercise	
	PRE	P0	PRE	P0
**EG**	2.3 ± 1.1	15.3 ± 3.5**	2.4 ± 0.9	14.9 ± 3.1**
**CG**	2.6 ± 0.9	14.9 ± 3.3**	2.5 ± 1.1	15.1 ± 3.5**
**Main effect - Time**	*p* = 0.886; η^2^ = 0.003		*p* = 0.918; η^2^ = 0.002	
**Main effect - Group**	*p* = 0.000; η^2^ = 0.962		*p* = 0.000; η^2^ = 0.974	
**Interaction - Time × Group**	*p* = 0.540; η^2^ = 0.056		*p* = 0.895; η^2^ = 0.003	

CG: control group; EG: experimental group. **p < 0.01 vs. PRE

### 3.3 Effects on Serum Cortisol, ACTH, SOD and WBC

**FIGURE 4:**
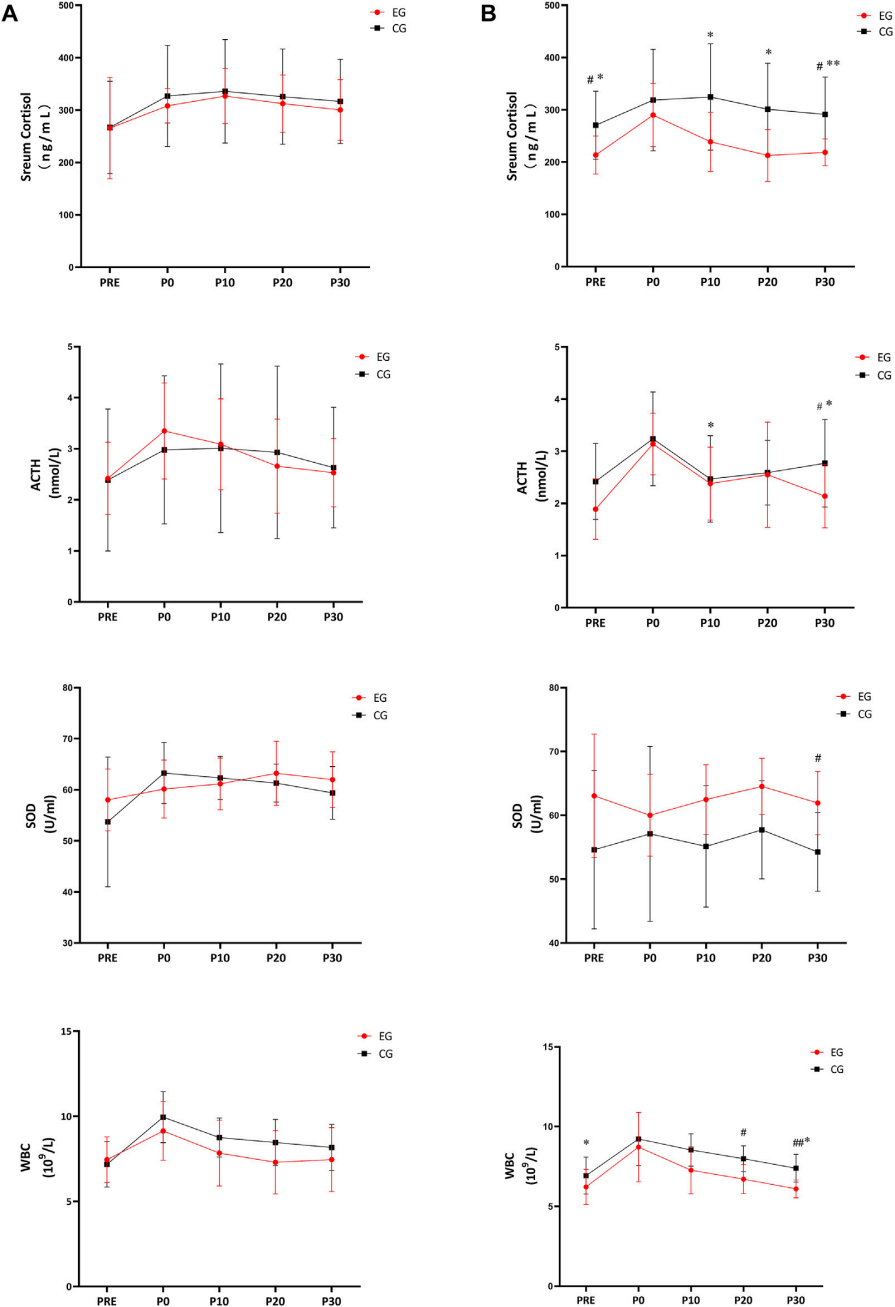
Effects of 4-week tangeretin supplementation and high-intensity resistance exercise on serum cortisol, ACTH, SOD and WBC. Notes: **(A)** first high-intensity resistance exercise; **(B)** second high-intensity resistance exercise; CG: control group; EG: experimental group; #*p* < 0.05, EG vs CG; ##*p* < 0.01, EG vs CG; *p < 0.05, vs. PRE; **p < 0.01 vs PRE.

For the first resistance exercise sessions (before the 4-week intervention), no significant difference was observed in the biochemical indices (serum cortisol, SOD and WBC) in EG and CG (A in Figure 4). After the 4-week intervention, the serum cortisol level of EG was significantly lower at PRE (*p* = 0.017), P10 (*p* = 0.010), P20 (*p* = 0.014), and P30 (*p* = 0.007) of the later resistance exercise sessions, compared to the values of the first resistance exercise sessions before intervention. The serum cortisol level of EG was also significantly lower than CG at PRE (*p* = 0.036) and P30 (*p* = 0.031) (Figure 4 serum cortisol-A). Similarly, ACTH decreases significantly in EG at P10 (*p* = 0.037) and P30 (*p* = 0.049), compared to pre-intervention values, and ACTH concentration of EG was significantly lower than that of CG at P30 (*p* = 0.044) (Figure 4 ACTH-B). The SOD activity of EG was significantly higher than that of CG in each time points after the intervention, with a significant drop at PRE (*p* = 0.037) and P30 (*p* = 0.046) throughout the provocation test (Figure 4 SOD-B). The SOD activity was significantly higher after intervention in CG at P30 (*p* = 0.003) than that in EG (Figure 4 SOD-A). Figure 4 WBC-B showed the WBC of the EG decreased significantly (PRE, *p* = 0.037; P30, *p* = 0.046) and was significantly lower than that of the CG (P20, *p* = 0.01; P30, *p* = 0.003).

## 4 Discussion

This study aimed to investigate the effect of 4-week tangeretin supplementation on the cortisol stress response triggered by high-intensity resistance exercise. We found that the serum cortisol level of athletes in EG decreased significantly after taking a 200 mg/day tangeretin (TG) supplement for 4 weeks. This result supports the initial hypothesis of the study: a significant reduction in serum cortisol level was also observed after the provocation test of high-intensity resistance exercises (shoulder press, back squat, bench press, and deadlift). The level of serum cortisol in EG is also lower compared with the CG. Other than serum cortisol, it was also noticed in this study that after TG supplementation, ACTH and WBC levels in the EG were reduced substantially and were lower than in the CG. SOD activity was higher in the EG than in the CG. These results suggest that TG supplementation during high-intensity resistance exercise helps to regulate cortisol stress response in the human body, suppress the excessive synthesis and secretion of cortisol, and improve the resilience against oxidative stress of the whole body. TG also helps the attenuation of inflammation response.

High-intensity resistance exercise is effective at triggering a cortisol stress response in humans, resulting in a rapid rise in cortisol levels (Kraemer et al., 2005; Wang et al., 2019; Tsuda et al., 2020). Compared to other types of stimulation such as fear and visual stimulation, high-intensity resistance exercise is more controllable, repeatable and non-invasive. It is the preferred method for studying the inherent relationship between external stimulation and the cortisol stress response (Finke et al., 2018; Wang et al., 2019). According to Kraemer et al. (2005), the four resistance exercise sessions, i.e., shoulder press, back squat, bench press, and deadlift, could effectively activate all major muscle groups of the human body quickly and trigger the optimum cortisol stress response when the workload is four sets × 4 repetitions × 10 RM (with a 2-min rest between sets and repetitions). Based on the above findings (Kraemer et al., 2005), the same resistance exercise regimen and workload are adopted in this study. Studies have found that after high-intensity resistance exercise, serum cortisol level of both EG and CG rises significantly, as does the blood lactate level, with an increase >14 mmol/L in both cases (Kraemer et al., 2005). These results show that the high-intensity resistance exercise stimulation performed here could effectively provoke cortisol stress response of human body, and the aforementioned exercise model can be used to investigate the effect of TG on the human cortisol stress response.

Flavonoids can effectively modulate the cortisol stress response and inhibit cortisol synthesis and secretion (Ohno et al., 2002; Loerz and Maser, 2017). In 2016, Kuebler found that the cortisol stress response in healthy participants was effectively suppressed by ingesting 50 g dark chocolate (containing 600 mg of total flavonoids) 2 h before the Trier Social Stress Test. A significant decline in serum cortisol level was also observed. Kraemer et al. (2005) also reported that a 4-week intervention with a flavonoid supplement mixture significantly alleviated the cortisol stress response triggered by high-intensity resistance exercise and accelerates the post-exercise recovery of serum cortisol level, which may be due to quercetin (a polymethoxyflavones) in the supplement inhibiting the conversion of 11-dehydro-17-hydroxycortisone to cortisol in adrenal cells. Quercetin and TG are both members of the same PMH flavonoid subfamily (Zhao et al., 2018). Preliminary studies by our research group have shown the positive regulatory role played by TG on cortisol. For example, after oral ingestion of 200 mg/d TG for 5 weeks, a significant decrease was seen in the level of fasting serum cortisol in the morning (Liu et al., 2019). Based on these results, our study further delineates the effect of 4-week TG supplementation on the cortisol stress response. After a 200 mg/d tangeretin supplementation intervention for 4 weeks, the serum cortisol level of the EG decreased significantly at 10 min (P10), 20 min (P20), and 30 min (P30) following provocation test. The cortisol level in the EG was significantly lower than that of the CG at P30. This suggested TG can lower the level of serum cortisol in the resting state and relieve the cortisol stress response induced by high-intensity resistance exercise. These findings lay the foundation for the application of nutritional supplements that provide rapid recovery from physical fatigue and inhibition of protein catabolism caused by high-intensity exercise.

The regulatory effect of flavonoids on the human cortisol stress response is also seen with ACTH, the key regulator of cortisol secretion and synthesis. When the body is fighting stress, ACTH secretion is accelerated in the hypothalamus, promoting the synthesis and secretion of cortisol in large quantities (Kuebler et al., 2016). An et al. reported a significant drop in ACTH and serum cortisol levels in experimental rats fed with the Heart Nourishing Tonic (total flavonoids content: 50 mg/kg) for 4 weeks (An et al., 2008). The researchers posited that flavonoids reduce human cortisol levels by inhibiting ACTH secretion. Kraemer et al. conducted provocation test with high-intensity resistance exercises to study the effect of flavonoid supplement mixtures containing quercetin on cortisol stress response (Kraemer et al., 2005). They believed that, after oral ingestion of the flavonoid supplement mixture for 4 weeks, the experimental group showed a significantly lower ACTH level than the control group at corresponding time points. At 5 min and 10 min after the provocation test, the ACTH level in the experimental group was also substantially lower than at pre-intervention times. The decrease in ACTH levels was shown to correspond well with the decrease in serum cortisol levels. Similar results were observed in our study. After 4 weeks of TG supplementation, a significant decrease in ACTH level was observed in EG at 10 min (P10) and 30 min (P30) after the stimulation experiment. Moreover, the ACTH level in EG was significantly lower than that of the CG at P30, which was consistent with the variation trend of serum cortisol level.

The human adrenocortical tumor cell line H295R is an ideal candidate for studying cortisol synthesis and secretion (Bertazza et al., 2019). Through *in vitro* study, Li et al. found that 24 h after the addition of 30 μM/unit of quercetin to H295R cell cultures, the expression of 11β-hydroxysteroid dehydrogenase (11-βHSD) mRNA decreased by 50% (Cheng and Li, 2012). They believe quercetin effectively limits the gene expression of 11-βHSD and in turn inhibits the conversion of 11-dehydro-17-hydroxycortisone (cortisone) to cortisol. Through forskolin stimulation (Hasegawa et al., 2013), found that addition of 30 μM naringin or 10 μM hesperetin to each unit of an H295R cell culture effectively reduces the activity of 3β-hydroxysteroid dehydrogenase (3β-HSD) and significantly lowers the level of cortisone. Moreover, Ohno et al. (2002) discovered that the mono-hydroxy flavone M6 (IC50 = 0.5–2.7 μM) could inhibit the activity of cytochrome P450 enzymes and 3β-HSD, thus reducing the synthesis and secretion of cortisol in H295R cells. TG and the four active compounds mentioned above (quercetin, naringin, hesperetin, and mono-hydroxy flavone M6) are all polymethoxyflavones (PMFs) with highly similar chemical structures and biochemical activities (Zhao et al., 2018). Therefore, we speculate that TG modulates the cortisol stress response by attenuating the gene expression of 11-βHSD and the activity of cytochrome P450 enzymes and 3β-HSD. This postulate, however, remains to be proven.

Supplementation with naturally derived antioxidants could effectively clear the large quantity of free radicals produced during high-intensity exercises, accelerating fatigue recovery and reducing serum cortisol levels (Kraemer et al., 2005; Braakhuis and Hopkins, 2015; Wu et al., 2019). TG is a naturally occurring antioxidant found in fruit and vegetables with three verified mechanisms of antioxidant activity. Results from chemical assays have shown that TG can effectively scavenge the free radicals of diammonium 2,2′-azino-bis (3-ethylbenzothiazoline-6-sulfonate (ABTS+•) and 1,1-diphenyl-2-trinitrophenylhydrazine (DPPH•), with scavenging rates of 8 and 10%, respectively (Tarozzi et al., 2006). One study based on cell models showed that TG significantly reduces the oxidative damage on HepG2 cells induced by tert-butylhydroperoxide (t-BHP) and enhances free radical scavenging efficiency (Selmi et al., 2017). Studies based on animal models (Kou et al., 2018; 2019) suggest that TG supplementation for 4 weeks (50 mg/d/kg) significantly increases antioxidant enzyme activity in mice and substantially alleviates the myocardial and skeletal muscle injury caused by oxidative stress from high-intensity, exhaustive exercise. In this study, we found that after 4 weeks of TG supplementation, the SOD activity of the EG remained stable during high-intensity resistance exercise, while that of the CG decreased gradually and was significantly lower than in the test group at corresponding time points. This study further confirms the high antioxidant capacity of TG in humans. Based on these results, we conclude that TG supplementation could boost antioxidant activity in humans and indirectly inhibit the synthesis and secretion of cortisol.

The strong stimulation of high-intensity resistance triggers a series of immune response changes in human skeletal muscles and other organs and leads to a sharp increase in the level of inflammatory factors, such as WBC and neutrophils, in the blood (Szlezak et al., 2016). We found that after two stimulation experiments via resistance exercise, the WBC count of all athletes showed a significant increase at P0, and then decreased gradually. After the second exercise stimulation experiment, the percent decline of WBC in the EG was significantly higher than that of the CG. This is thought to be related to the anti-inflammatory and immunomodulatory activities of TG. Molecular studies have shown that TG could significantly lower the level of pro-inflammatory cytokine TNF-α and enhance the activity of anti-inflammatory cytokine IL-1α (Arab et al., 2016). Animal studies have shown that a 14-day TG intervention (25 mg/kg/day) significantly reduces the level of inflammatory cytokines such as Th2 and Th17 in P12 mice and effectively inhibits the bronchitis inflammation induced by exhaustive exercise (Liu et al., 2017). Previous studies (Liu et al., 2019) suggest that after oral administration of TG (200 mg/day) for 5 weeks, joint and muscle pain and the risk of acute muscle strain both decrease slightly during high-intensity exercise in weightlifters and WBC count level decreases significantly on the next morning. Some studies have reported the anti-inflammatory, anti-stress, and immune hypersensitivity-inhibiting functions of cortisol (Fantidis, 2010; Walter et al., 2018). This compound is thus important in eliminating inflammation and repairing muscle after exercise. However, our study showed that a 4-week TG supplementation regimen does not damage the overall anti-inflammatory function of the body even though the WBC count was significantly reduced before and after high-intensity resistance exercise. This means taking TG supplements for 4 weeks may facilitate the repair and regeneration of muscle and other tissues, and indirectly promote functional recovery. However, the internal mechanism of these activities requires further research for revelation.

## 5 Conclusion

This study explored the effect of tangeretin supplementation on the cortisol stress response stimulated by high-intensity resistance exercise. We found that a 4-week tangeretin supplementation (200 mg/day) effectively reduces the cortisol stress response triggered by high-intensity resistance exercise, reduces serum cortisol and ACTH levels, and enhances the resilience of the body toward oxidative stress and inflammation. No adverse physiological or emotional reactions such as insomnia, nausea, or irritability were observed in the athletes during the period of tangeretin supplementation. Thus, tangeretin can be used to as a promising sports supplements to achieve a specific physical performance or health benefit in training and competitions, especially for high-intensity resistance exercises such as sprinting and weightlifting.

## Data Availability

The raw data supporting the conclusions of this article will be made available by the authors, without undue reservation.
