# Supplementary material for: Effects of 4-Week Tangeretin Supplementation on Cortisol Stress Response Induced by High-Intensity Resistance Exercise: A Randomized Controlled Trial
Source: Front Physiol. 2022 May 19;13:886254. doi: 10.3389/fphys.2022.886254 (PMC9160924; doi:10.3389/fphys.2022.886254)
Supplement: Supplementary file 1 [file Table1.DOCX]

|  | **Body Weight (kg)** | | **Body Fat Percentage (%)** | | **Muscle Mass (kg)** | |
| --- | --- | --- | --- | --- | --- | --- |
|  | R1 | R2 | R1 | R2 | R1 | R2 |
| **EG** | 61.8 ± 6.0 | 61.9 ± 5.9 | 13.3 ± 4.4 | 12.9 ± 3.7 | 30.5 ± 4.3 | 30.8 ± 4.3 |
| **CG** | 59.1 ± 9.8 | 59.3 ± 10.3 | 12.9 ± 4.7 | 13.1 ± 4.6 | 28.6 ± 5.3 | 28.9 ± 5.7 |
| **Main effect - Time** | *P* = 0.408; η²=0.063 | | *P* = 0.665; η²=0.020 | | *P* = 0.353; η²=0.087 | |
| **Main effect - Group** | *P* = 0.448; η²=0.053 | | *P* = 0.459; η²=0.056 | | *P* = 0.050; η²=0.332 | |
| **Interaction - Time× Group** | *P* = 0.921; η²=0.001 | | *P* = 0.115; η²=0.229 | | *P* = 0.869; η²=0.003 | |
| CG: control group; EG: experimental group. | | | | | | |

**Table 1. Effects of Tangeretin Intervention on Body Composition**
