# Supplementary material for: Effects of 4-Week Tangeretin Supplementation on Cortisol Stress Response Induced by High-Intensity Resistance Exercise: A Randomized Controlled Trial
Source: Front Physiol. 2022 May 19;13:886254. doi: 10.3389/fphys.2022.886254 (PMC9160924; doi:10.3389/fphys.2022.886254)
Supplement: Supplementary file 2 [file Table2.DOCX]

**Table 2. Comparison of 10RM of the back squat, bench press, deadlift, and shoulder press (kg)**

|  | **Shoulder Press（kg）** | | **Back Squat（kg）** | | **Bench Press（kg）** | | **Deadlift（kg）** | |
| --- | --- | --- | --- | --- | --- | --- | --- | --- |
|  | T1 | T2 | T1 | T2 | T1 | T2 | T1 | T2 |
| **EG** | 54.3 ± 19.1 | 58.2 ± 21.8 | 91.1 ± 22.4 | 93.2 ± 23.5 | 49.6 ± 17.2 | 52.9 ± 16.4 | 93.2 ± 22.7 | 94.3 ± 23.2 |
| **CG** | 53.6 ± 18.2 | 54.6 ± 17.9 | 89.6 ± 21.7 | 92.1 ± 22.3 | 48.2 ± 16.2 | 50.7 ± 15.1 | 95.4 ± 23.6 | 97.1 ± 25.2 |
| **Main effect - Time** | P = 0.396; η²=0.056 | | P = 0.110; η²=0.184 | | P = 0.065; η²=0.238 | | P = 0.518; η²=0.033 | |
| **Main effect - Group** | P = 0.029; η²=0.318 | | P = 0.138; η²=0.161 | | P = 0.001; η²=0.610 | | P = 0.055; η²=0.254 | |
| **Interaction - Time × Group** | P = 0.241; η²=0.104 | | P = 0.583; η²=0.024 | | P = 0.165; η²=0.143 | | P = 0.612; η²=0.020 | |
| CG: control group; EG: experimental group. T1: Before 4-week tangeretin intervention; T2: After 4-week tangeretin intervention. | | | | | | | | |
