# Supplementary material for: Effects of 4-Week Tangeretin Supplementation on Cortisol Stress Response Induced by High-Intensity Resistance Exercise: A Randomized Controlled Trial
Source: Front Physiol. 2022 May 19;13:886254. doi: 10.3389/fphys.2022.886254 (PMC9160924; doi:10.3389/fphys.2022.886254)
Supplement: Supplementary file 3 [file Table3.DOCX]

**Table 3. Comparison of blood lactate before and after high-intensity resistance exercise test (mmol/L)**

|  | **1st high-intensity resistance exercise** | | **2nd high-intensity resistance exercise** | |
| --- | --- | --- | --- | --- |
|  | PRE | P0 | PRE | P0 |
| **EG** | 2.3 ± 1.1 | 15.3 ± 3.5** | 2.4 ± 0.9 | 14.9 ± 3.1** |
| **CG** | 2.6 ± 0.9 | 14.9 ± 3.3** | 2.5 ± 1.1 | 15.1 ± 3.5** |
| **Main effect - Time** | P = 0.886; η²=0.003 | | P = 0.918; η²=0.002 | |
| **Main effect - Group** | P = 0.000; η²=0.962 | | P = 0.000; η²=0.974 | |
| **Interaction - Time × Group** | P = 0.540; η²=0.056 | | P = 0.895; η²=0.003 | |
| CG: control group; EG: experimental group. ** P < 0.01 vs PRE. | | | | |
