# Supplementary material for: Effects of 4-Week Tangeretin Supplementation on Cortisol Stress Response Induced by High-Intensity Resistance Exercise: A Randomized Controlled Trial
Source: Front Physiol. 2022 May 19;13:886254. doi: 10.3389/fphys.2022.886254 (PMC9160924; doi:10.3389/fphys.2022.886254)
Supplement: Supplementary file 4 [file DataSheet2.DOCX]

|  | pre | p0 | p10 | p20 | p30 |
| --- | --- | --- | --- | --- | --- |
| Female In EG | 268.45 ±80.03 | 310.50 ±43.08 | 309.00 ±39.45 | 284.71 ±46.92 | 258.70 ±36.28 |
| Male In EG | 263.11 ±135.84 | 305.69 ±31.61 | 344.74 ±70.28 | 339.95 ±60.99 | 339.67 ±56.63 |
| Female In CG | 252.38 ±99.60 | 307.24 ±55.05 | 333.93 ±51.13 | 320.74 ±77.00 | 304.40 ±83.76 |
| Male In CG | 281.52 ±101.18 | 346.57 ±144.28 | 337.84 ±170.15 | 330.55 ±126.55 | 336.29 ±110.86 |
| *P* value | 0.984 | 0.875 | 0.957 | 0.800 | 0.448 |
|  |  |  |  |  |  |
| **Effects of 4-Week Tangeretin Supplementation and the 2nd High-Intensity Resistance Exercise on Cortisol in different genders and groups** | | | | | |
|  | pre | p0 | p10 | p20 | p30 |
| Female In EG | 192.54 ±31.22 | 298.16 ±127.26 | 232.42 ±35.63 | 203.73 ±46.18 | 196.72 ±18.79 |
| Male In EG | 241.88 ±67.71 | 264.98 ±79.82 | 275.81 ±45.25 | 259.52 ±39.88 | 257.81 ±47.08 |
| Female In CG | 264.06 ±58.29 | 286.96 ±45.27 | 267.32 ±31.35 | 246.28 ±19.13 | 235.82 ±2.77 |
| Male In CG | 277.26 ±88.72 | 330.28 ±73.69 | 301.76 ±53.41 | 272.20 ±44.34 | 326.66 ±166.11 |
| *P* value | 0.314 | 0.760 | 0.195 | 0.125 | 0.247 |

**Effects of 4-Week Tangeretin Supplementation and the 1st High-Intensity Resistance Exercise on Cortisol in different genders and groups**
