# Supplementary material for: Effects of 4-Week Tangeretin Supplementation on Cortisol Stress Response Induced by High-Intensity Resistance Exercise: A Randomized Controlled Trial
Source: Front Physiol. 2022 May 19;13:886254. doi: 10.3389/fphys.2022.886254 (PMC9160924; doi:10.3389/fphys.2022.886254)
Supplement: Supplementary file 5 [file DataSheet1.DOCX]

|  | **Weekly Training Plan(week×4)** | | | | | |
| --- | --- | --- | --- | --- | --- | --- |
|  | **Monday** | **Tuesday** | **Wednesday** | **Thursday** | **Friday** | **Saturday** |
| **AM**  **9:00-11:30** | **Preparation activities**  Stretching Exercises  Agility exercises  **4 v 4 Goal Support**  (Pitch size:30×20 m  3×6min,1.30 min rest  HR 85%max)  **10 v 10 match**  60 min | **Preparation activities**  Stretching Exercises  ball familinarity  **4 v 4 side support**  (Pitch size:50×40 m  4×4min,  3 min active rest,  3×8min,  2 min active rest,  HR 85-90%max)  **10 v 10 match**  60 min | **Preparation activities**  Stretching Exercises Agility exercises  **5 v 5 game**  (Pitch size:50×40 m  2×10min,  3 min active rest,  2×10min,  2 min active rest,  HR 85-90%max)  **10 v 10 match**  60 min | **Preparation activities**  ball familinarity  **4 v 4 Goal Support**  (Pitch size:30×20 m  3×6min,1.30 min rest  HR 85%max)  **Bodyweight workouts**  upper body  core  lower body  **10 v 10 match**  60 min | **Preparation activities**  Stretching Exercises Agility exercises  **4 v 4 side support**  (Pitch size:50×40 m  4×4min,  3 min active rest,  3×8min,  2 min active rest,  HR 85-90%max)  **10 v 10 match**  60 min | **Preparation activities**  Stretching Exercises  Agility exercises  **11 v 11 game**  90 min |
| **PM**  **3:00-5:30** | **Barbell CMJ Jump**  (15-20kg,4×4)  **Squat 3RM** (3×3)  **Bench Press 3RM** (3×3)  **Barbell Recumbent 3RM** (3×3) | **Medicineball workouts**  upper body  core  lower body | **Barbell CMJ Jump**  (10kg,4×10)  **Squat 10RM** (3×10)  **Bench Press 10RM** (3×10)  **Barbell Recumbent 1RM** (3×1) | Soccer Tactical Analysis | **Barbell CMJ Jump**  (10kg,4×10)  **Squat 10RM** (3×10)  **Bench Press 10RM** (3×10)  **Barbell Recumbent 1RM** (3×1) | Soccer Tactical Analysis |
| All athletes rest on Sunday | | | | | | |

**Training Content, Quantity, and Intensity**
